# On cell loss in Parkinson’s disease, and the citations that followed

**DOI:** 10.1038/s41531-022-00306-x

**Published:** 2022-04-11

**Authors:** Samuel Burke, Louis-Eric Trudeau

**Affiliations:** 1 Department of Pharmacology and Physiology, Faculty of Medicine, Université de Montréal, Montréal, QC Canada; 2 Department of Neurosciences, Faculty of Medicine, Université de Montréal, Montréal, QC Canada; 3 Neural Signaling and Circuitry Research Group (SNC), Université de Montréal, Montréal, QC Canada

**Keywords:** Parkinson's disease, Parkinson's disease

Writ large, Parkinson’s disease (PD) is caused by the dysfunction and subsequent loss of several neuronal populations, notably dopamine neurons of the Substantia Nigra pars compacta (SNpc). Identifying the neurochemical and neuroanatomical identity of these neuronal populations—as well as the temporal order of their degeneration—is fundamental to understanding this disease.

The fundamental work by Braak et al.^1^ as well as that of other teams, has led to a gradually increased understanding of the spatial and temporal development of Lewy pathology in PD, as well as dementia with Lewy bodies. Although this Lewy pathology is likely to be crucial in explaining neuronal loss, it appears that many claims concerning the spatiotemporal pattern of neuronal loss in PD are based on Lewy pathology, rather than overt cell loss. Counting neurons in post-mortem tissue is the only way to generate the data that is needed to understand properly such spatiotemporal pattern underlying the multiple symptoms of this disease. And the most (and only) reliable method for this is unbiased design-based stereology^2–4^. Stereology is a method for “neuromorphometry” that has been around for well over a century^5^, however, its application to the SNpc is comparatively recent^6^, and much of the data on cell loss in PD precedes its widespread implementation. Not only has large parts of the literature on cell loss in PD not used design-based stereology, but much has also not used simple standards of blinding and reporting essential clinical variables, let alone comparing cell counts of multiple neuronal populations in the same cases^7^.

In 2018 we published a review article^7^ where we aimed to communicate that, given the available literature, there is insufficient evidence to support granular claims made about the specific neurochemical identity of cells lost in the brain of people diagnosed with PD and to make conclusive and broad statements about the temporal order of this specific cell loss. Our goal was also to encourage the field to undertake further quantitative studies on neuronal loss in the human brain in PD, comparing multiple brain regions.

This review has gone on to be well-cited (2021-09-24: Google Scholar: 136), yet, despite our presented data and arguments, statements citing this work very often do not integrate the view we have put forward. In fact, several even cite this piece in support of what is now most probably a dogma. Motivated by this observation, in this letter we aim to draw to the attention of basic and clinical scientists working on PD that the identity and temporal order of neuronal degeneration in PD is still an open question. Given that the specific identity and temporal order of degeneration underpin hypotheses of selective vulnerability—shaping the nature of comparative work trying to distinguish the features of neurons that render them vulnerable in PD and the paradigms that major funding bodies are supporting—we believe it essential that new longitudinal studies be conducted addressing these questions.

Our aim here is to illustrate and understand the nature in which our previous review has impacted our field’s understanding of PD. We note that most papers that cited our work, did so in support of statements on general background information related to PD, and PD pathophysiology. However, we have noticed that very few claims integrate our core message: that as a field, we need to generate better quality data on the location and order of neuronal degeneration in PD.

Of the 136 citations listed by Google Scholar (the highest count across indexing platforms), we extracted 153 statements from 114 scientific documents citing our review (written in English and from peer-reviewed articles or theses). We then characterized these statements according to the criteria listed in Table 1.

**Table 1:** Statements made citing our review categorized according to the described criteria.

Classification of claims citing our review	
Classification description	Instances
**Statements addressing broad background information on PD**. Broad information on PD. e.g., PD is associated with loss of dopamine (DA) neurons of the Substantia Nigra pars compacta.	**124**
**Statements addressing core message of review**. Referring to the lack of data on the spatiotemporal pattern of PD-specific cell loss.	**7**
**Statements made that are contrary to contents of review**. Inaccurate given the contents of our review. e.g. Ventral Tegmental Area dopamine neurons are unaffected in PD.	**7**
**Statements that are citing specific data from within the review**. Refers to specific data available from a study cited within our review.	**39**
**Statements made that are unrelated to review, or inaccurate**. Claiming specific loss, or lack of loss of neuronal types not mentioned in review.	**12**

Multiple statements made within a single publication were categorized individually.

The scarcity of statements made citing our core message suggests that our field is yet to integrate this message into its understanding of cell loss in PD. We observe that an equal number of statements (seven) are made addressing the core message of the review, as those that are inaccurate (continuing a narrative that this spatiotemporal pattern is well-established). Considering that how we cite can impact scientific narratives for decades^8,9^, we hope that by addressing the nature of how the research community has taken up our initial message, we can contribute to accelerating progress in our field: avoiding epistemic cul-de-sacs driven by our research culture^10^, and re-opening avenues towards progress.

We must highlight a notable exception by Huynh and colleagues^11^—whether impelled or not by our work—re-examined previous cases of a “well-characterized cohort”. Acquiring bilateral brain stem samples (with detailed clinical records) from a previous study^12^, Huynh et al. performed the first stereological quantifications of noradrenergic locus coeruleus neurons in confirmed cases of PD. Promisingly, in line with previous quantifications and with models proposing increased vulnerability of long-range projection neurons, they find substantial neuronal loss with ~43% of noradrenergic neurons remaining in this structure for cases with PD, and ~14% for cases of PD with dementia, compared to controls (with an *n* of 9, 5, and 7, respectively).

Notwithstanding, this study is unique, and it remains essential that we acquire more extensive longitudinal data on multiple neuronal populations, in the same cases, with detailed clinical variables measured. Furthermore, given the absence of such data in other neurogenerative diseases, and considering that, for example, the loss of SNpc dopamine neurons is also an important variable in the diagnosis of Dementia with Lewy Bodies^13^, it would clearly be a worthy investment to extend such work across disease labels.

At the time of writing, we have not received correspondence suggesting that we have missed vital literature, nor that our claims are unwarranted. Nevertheless, we have come to realize that literature was missed. With colleagues, a preregistered systematic review and meta-analysis^14^ is ongoing to quantify cell loss in confirmed cases of PD across all studies available.

The following quote we believe embodies the urgency of addressing the points we raise: “What if our understanding of PD is also impeding our ability to find cures? Could it be that generating hypotheses based on what we think we know, along with our rigid funding models, is making it nearly impossible to find what we really need to know?”^15^. As scientists, we must continue to serve the individuals at the end of our label—those diagnosed with PD, today and in the future.

Supplementary table (containing the list of citing works, claims made, and characterization) available from the corresponding author on reasonable request.

## Reporting summary

Further information on research design is available in the Nature Research Reporting Summary linked to this article.

## Supplementary information

Reporting Summary
